# Selective Spleen Scintigraphy in the Evaluation of Accessory Spleen/Splenosis in Splenectomized/Nonsplenectomized Patients and the Contribution of SPECT Imaging

**DOI:** 10.4274/mirt.40085

**Published:** 2015-02-15

**Authors:** Şeyma Ekmekçi, Reyhan Diz-Küçükkaya, Cüneyt Türkmen, Işık Adalet

**Affiliations:** 1 İstanbul University Faculty of Medicine, Department of Nuclear Medicine, İstanbul, Turkey; 2 İstanbul University Faculty of Medicine, Department of Internal Medicine, Division of Hematology, İstanbul, Turkey

**Keywords:** Spleen, splenosis, scintigraphy, single-photon emission-computed tomography

## Abstract

**Objective:** We aimed to evaluate the results of selective spleen scintigraphy (SSS) and contribution of SPECT imaging to planar imaging in splenectomized and nonsplenectomized patients.

**Methods:** We retrospectively examined 112 SSSs of 96 patients. The patients were divided into two groups as splenectomized group (SP) and non-splenectomized group (NSP). The findings were evaluated by comparing the results of surgery,computerized tomography (CT), ultrasonography (USG) and magnetic resonance imaging (MRI). In addition, whether or not differences existed between the results of SPECT and planar imaging was determined.

**Results:** Of 66 scintigraphies performed in the NSP group, 3 (5%) had positive, 3 (5%) had suspicious and 60 (90%) had negative results. In the NSP group, 28 patients underwent surgery and 12 accessory spleens were removed. Only 3 of these tissues were detected by scintigraphy. Of 46 patients in the SP group, 26 (57%) had positive findings whereas 20 (43%) had negative scintigraphies. Twelve accessory spleens/splenosis were removed surgically in 10 patients with a positive SSS in the SP group. There were no false positive results in both groups of patients who underwent surgery. There was no significant difference between the results of SSS, USG and CT. Of 39 patients to whom SPECT were performed, 10 had positive results both with planar and SPECT imaging. On the other hand, 26 patients, 3 of whom had suspected findings in SPECT images, demonstrated negative results when evaluated with both imaging methods. Remaining 3 were considered suspicious by only SPECT images for the hilar area.

**Conclusion:** SSS has high specificity in the detection of accessory spleens/splenosis. The sensitivity of SSS is low in the NSP group,but higher in the SP group. There is no contribution of SPECT imaging to planar imaging.

## Abstract (tr)

**Amaç:** Çalışmamızda, splenektomize ve non-splenektomize hasta gruplarında selektif dalak sintigrafisinin (SDS) sonuçlarını ve SPECT görüntülemenin planar görüntülemeye katkısını incelemeyi amaçladık.

**Yöntem:** Doksan altı hastada yapılmış 112 SDS retrospektif olarak incelendi. Hastalar splenektomize (SP) ve non-splenektomize (NSP) olmak üzere 2 gruba ayrıldılar. Sintigrafi sonuçları hastaların ameliyat bulguları, bilgisayarlı tomografi (BT), ultrasonografi (USG) ve manyetik resonans görüntüleme (MRG) sonuçları ile karşılaştırıldı. Ayrıca SPECT görüntülemenin planar görüntülemeye katkısı olup olmadığı değerlendirildi.

**Bulgular:** NSP grubunda yapılan 66 sintigrafinin 3’ünde (%5) sonuç pozitif iken, 3’ünde (%5) şüpheli, 60’ında (%90) ise negatifti. Bu grupta 28 hasta opere edildi ve 12 aksesuar dalak çıkarıldı. Bu olguların yalnız 3’ü sintigrafik olarak saptanabilmişti. SP grubu oluşturan ve nüks görülen 46 hastanın 26’sında (%57) SDS pozitif iken, 20’si (%43) negatifti. Her 2 grupta ameliyat olan hastaların hiçbirinde sintigrafi sonuçları yalancı pozitif değildi. SDS, USG ve BT sonuçları arasında istatistiksel açıdan anlamlı fark yoktu. SPECT yapılan 39 hastanın 10’unda hem planar, hem de SPECT görüntüler ile aksesuar dalak saptanırken, 26’sında her iki metodla da aksesuar dalak tespit edilmemiş, 3 hastada sonuçlar yalnız SPECT görüntüler ile hiler bölge şüpheli olarak değerlendirilmiştir.

**Sonuç:** SDS’nin aksesuar dalak ve splenozis saptanmasındaki özgüllüğü yüksektir. Duyarlılığı NSP grubunda düşükken, SP grubunda daha yüksektir. SPECT görüntülemenin planar görüntülemeye ek bir katkısı saptanmamıştır.

## INTRODUCTION

An accessory spleen or splenosis is an important cause of recurrence in diseases in which splenectomy is curative, such as chronic immune thrombocytopenic purpura (crITP) and hereditary spherocytosis (HS) (1). Indeed, removal of the accessory spleen or spleen fragments is therapeutic for these diseases (2). In addition, splenosis may cause severe symptoms depending on the site of implantation (3,4,5). Splenosis may also be asymptomatic. However, when incidentally detected using radiologic methods, accessory spleen/splenosis may require differential diagnosis with recurrence or metastases in patients with malignancies (6,7,8). Accessory spleens that cannot be removed at the time of the initial splenectomy and splenosis may also be atypically located. Therefore, it is important to identify these tissues and establish an appropriate differential diagnosis.

The methods used in imaging include computed tomography (CT), ultrasonography (USG), magnetic resonance imaging (MRI) and scintigraphy. Selective spleen scintigraphy (SSS), which is performed using denatured erythrocytes labeled with Technetium-99m (Tc99m), is the preferred scintigraphic method due to the absence of liver uptake and increased specificity (9,10). The SPECT system, which enables scintigraphic images to be examined in tomographic cross-sections, has further improved the sensitivity of many scintigraphic examinations during the last 10 years and is now routinely used (11). Unfortunately, there are few comparative clinical studies in the literature other than case reports regarding the application of SPECT in SSS.

In the current study, we aimed to evaluate the results of SSS and contribution of SPECT imaging to planar imaging in splenectomized and nonsplenectomized patients.

## MATERIALS AND METHODS

One hundred twelve scintigraphies from 96 patients who had undergone SSS were retrospectively evaluated in the Department of Nuclear Medicine of Istanbul University Istanbul Faculty of Medicine between January 2003 and May 2009. The scintigraphies were divided into 2 following groups: splenectomized group (SP, n=46) and non-splenectomized group (NSP, n=66). The results of SSS were assessed and compared with the results obtained from surgery, CT, USG and MRI in both groups. Whether or not there were differences between the results of planar and SPECT imaging was determined.

Other imaging methods (USG, n=44; CT, n=31 and MRI, n=4) performed at the same time for the same purpose and discharge reports of surgery and pathology results for 38 patients were obtained from the files.

**Selective Spleen Scintigraphy Protocol**

One gr of stannous ion (Sn+2) in the form of pyrophosphate was injected intravenously to the patient. Approximately 20 min later, a blood sample (approximately 4 cc) was obtained, 20 mCi of NaTc99mO4 was added, the sample was incubated at 49 °C for 20 min, then the sample was re-injected to the patient. Scintigraphy was begun 20-30 min later. Anterior, posterior, left anterior oblique (LAO), left posterior oblique (LPO) and left lateral (LLAT) views of the abdomen are obtained with high-resolution low-energy collimators for 5 minutes on conventional gamma cameras. Sixty-four projections over 360 degrees (20 seconds per stop) were acquired. A reconstruction procedure was performed with back projection and an appropriate cut-off frequency of order was applied for filtering.

**Statistical Analysis**

No test which is accepted as gold standart exists that provides a comparison of the results for SSS. Surgical evaluation for the NSP group was limited to the area of surgery and is insufficient for unusual accessory spleen sites, such as the retroperitoneal region and the pelvis. Also in the SP group only the patients who had accessory spleens/splenosis based on imaging methods underwent surgery. Therefore, the sensitivity and specificity were not calculated, instead correlation tests were applied.

SPSS for Windows (version 10.0; SPSS, Inc., Chicago, IL, USA) was used. The McNemar test was used for comparisons and the Kappa test was used for consistency. Numeric data were expressed as the mean ± SD. P<0.05 was considered significant. Kappa coefficient ranges from 0.10-0.29, 0.30-0.49 and 0.5-1 were considered low, moderate and highly consistent, respectively.

The Ethics Committee of İstanbul University, İstanbul Faculty of Medicine approved the study protocol.

## RESULTS

Sixty-four patients were female and 32 were male (mean age, 29.5±20 years). Fifty-eight patients were referred with a diagnosis of crITP, 16 patients had HS, 1 patient had hereditary eliptositosis and 21 patients had other diagnoses. Of 112 scintigraphies, 46 were obtained in patients who had undergone previous splenectomy and 66 were performed in patients who had not yet undergone splenectomy. Of 112 SSSs, 29 were positive (26%), 80 were negative (71%) and 3 (3%) were suspicious for accessory spleens/splenosis. Two patients had 3 and 5 patients had 2 accessory spleens/splenosis detected; 18 patients had a single accessory spleen/splenosis detected. In addition, four patients had uptake which was suggestive of multiple splenosis (Table 1).

**Selective Spleen Scintigraphy Results in Non-Splenectomized Group**

Of 66 patients undergoing SSS prior to splenectomy, 3 (5%) were positive, 3 (5%) were suspicious and 60 (90%) were negative for an accessory spleen (Table 1).

**Figure 1:**
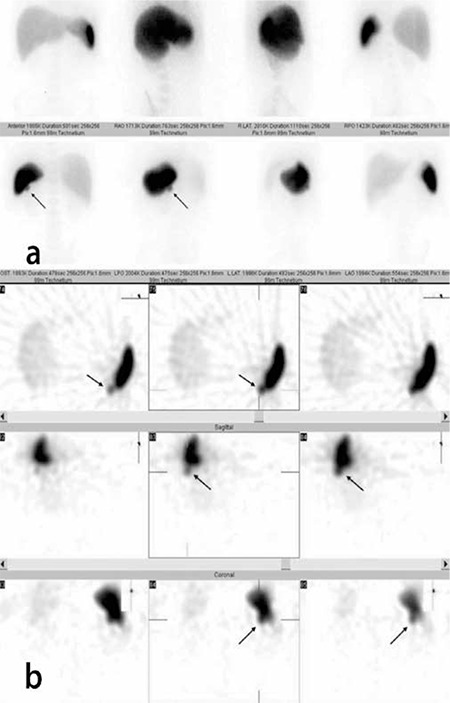
(a) 18-year-old male patient. He underwent SSS prior to splenectomy during follow-up for crITP. Increased focal uptake compatible with accessory spleen adjacent to the infero-posterior edge of the spleen (arrow). An accessory spleen 2x1, 5x1 cm in size was removed in the same location at the operation. (b) shows an uptake compatible with accessory spleen adjacent to the infero-posterior edge of the spleen in SPECT images (arrow)s

Accessory spleens were detected in the lower pole area and adjacent inferiorly to the lower pole and inferior edge of the spleen in non-splenectomized patients (Figure 1), while three suspected accessory spleens were thought to be localized in the hilar region. Of 66 patients in this group, 28 subsequently underwent splenectomies and 12 accessory spleens were removed from 9 patients (Table 2). Of these accessory spleens, 3 were detected by scintigraphy, 2 of which were 2 cm in size and 1 of which was 1 cm in size. Of 9 accessory spleens which were not detected by scintigraphy, 4 were located in the hilus and measured 0.5-1.5 cm in size. Other 5 accessory spleens were between 0.3-1 cm in size. Three patients with suspicious scintigraphy findings did not undergo surgery.

There was a significant difference between the results of scintigraphy and operative findings (p=0.031 (McNemar test)), and the findings were moderately consistent (kappa=0.40; p<0.008).

**Selective Spleen Scintigraphy Results in Splenectomized Group**

**Figure 2:**
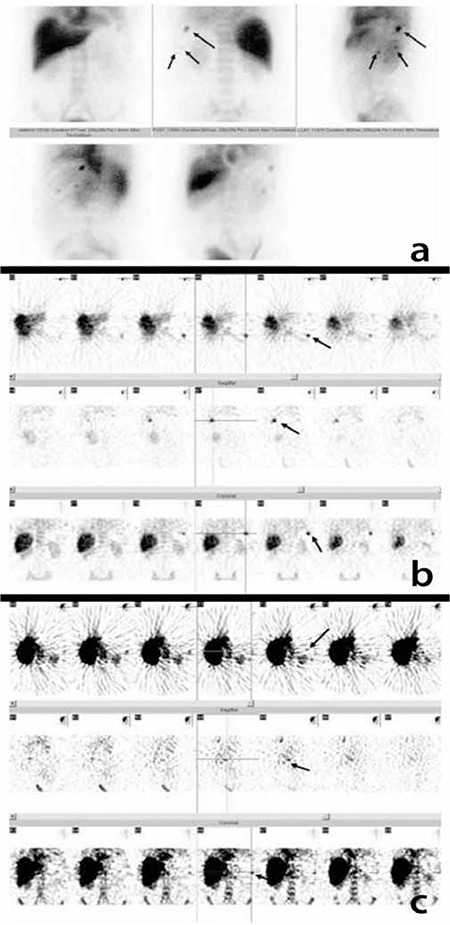
A 55-year-old male patient. He had undergone splenectomy with the diagnosis of crITP, but upon noting lower platelet values on follow-up, three foci were found in the spleen location on SSS performed for the suspicion of accessory spleen/splenosis (arrow) (2a planar, 2b and 2c SPECT images). An increase in platelet counts after the operation was observed

Of 46 SSS in the SP group, 20 (43%) were negative and 26 (57%) were positive. Twenty-two SSS had 31 focal and 4 patients had multiple small accessory spleens/splenosis (Figure 2) (Table 1). The localizations were as follows: spleen area (Figure 2), around the left kidney, stomach and its surroundings and the anterior left upper quadrant. In one patient with an history of traumatic rupture of the spleen, two focal lesions in the pelvic region were consistent with splenosis.

In this group, 10 patients with positive scintigraphies underwent re-operations and 12 accessory spleens/splenosis were removed. In one patient with one lesion determined by scintigraphy, three accessory spleens/splenosis localized very closely were removed and in one patient with two lesions, three accessory spleens/splenosis localized very closely were removed.

The pathology reports showed that the sizes of 10 lesions measured were 2.5 cm, 2 cm, 0.8x0.6x0.4 cm, 1.5x2.5x1 cm, 2x1.6x1.5 cm, 8x8 cm, 1.5 cm, 1.5 cm, 0.6 cm and 0.5 cm. In the pathology reports from two cases, no lesion sizes were specified. Comparison of SSS, USG, CT, and operation results in patients with positive scintigraphies is given in Table 3.

**Comparison of Selective Spleen Scintigraphy with Other Imaging Findings**

A patient with a positive USG, but with a negative scintigraphy did not undergo surgery. Accessory spleens/splenosis were removed from five patients with negative USGs, but with positive scintigraphies. A patient with a negative USG, but with a suspected focus in the splenic hilum on scintigraphy did not undergo surgery. There was no significant difference between the USG findings and the SSS results (p=0.219 (McNemar test)); however, moderate consistency existed (kappa=0.43; p<0.002).

An accessory spleen, 1.5 cm in size, was removed from the hilar region in a patient with a positive CT and a negative SSS. In comparing the CT findings and SSS results, there was no significant difference (p=1.000 (McNemar test)) and there was a very strong consistency (kappa=0.87; p<0.000).

Table 4 was formed on the basis of the patients. There are few differences in the results when evaluated on the basis of the lesions. A single bilobed mass was surgically removed from the patient in whom two lesions were demonstrated by USG and one lesion was detected by SSS. Similarly, three closely located lesions were removed surgically in a patient with four lesions detected on CT scan and two lesions detected on scintigraphy. Based on scintigraphy, three lesions in a patient were observed; the CT lesions were consistent with multiple splenosis in the area of the spleen and around the stomach. Based on MRI images of another patient, two adjacent lesions were observed measuring 1.5 and 0.7 cm in diameter; on scintigraphy a single focal uptake was noted in the superior upper pole of the left kidney. On MRI of another patient, multiple lesions were observed in the area of the spleen, while on scintigraphy two lesions were detected at the same site; however, one lesion was surgically removed.

**Comparison of SPECT Imaging with Planar Imaging**

When the findings from 39 patients who underwent planar and SPECT imaging were reviewed, 9 patients had 12 accessory spleens/splenosis identified in the same locations on the planar and SPECT images. Multiple small uptakes were detected in one patient using both techniques. In 26 patients, all of the results were negative using both methods. The results of SPECT images in three patients were considered to be suspicious in the hilar region, while the planar images were negative (Table 5). These three patients did not undergo surgery.

There was no difference between the results of SPECT and planar imaging p=1.000 (McNemar test), but there was full compliance with kappa (1 was determined), p<0.000; (Figure 1, 2).

## DISCUSSION

Accessory spleens are located in fatty tissue which has an attenuation coefficient different from that of accessory spleen. Also they have relatively specific localizations and standard morphology. Therefore, accessory spleens are easily recognized using radiologic imaging methods. However, splenosis becomes implanted to organs or their serous membranes which have similar attenuation coefficients, as well as similar contrast characteristics to splenosis. Also splenosis may be found in unusual locations. Thus it may be more difficult to diagnose splenosis (12,13). Indeed, a history of previous trauma and splenectomy is suggestive of the diagnosis, but the differential diagnosis requires more specific diagnostic tools.

Technically, the target-to-background ratio is high in SSS; however, high splenic uptake and superimposition of an accessory spleen may also be disadvantages. In patients who have undergone splenectomies, the blood pool activity is high due to decreased clearance of denatured red blood cells, thus the target-to-background ratio is lower.

As mentioned above, because accessory spleens/splenosis are different clinical situations and the presence of the spleen have a technical influence on SSS, we decided to evaluate the scintigraphies by dividing the patient cohort into two groups (before splenectomy and after splenectomy).

In the current study, the rate of detection of accessory spleens in the NSP group by SSS was 5%. The rate of diagnosing accessory spleens is 10-30% in the general population based on different studies in the literature (14). Thus, the sensitivity of SSS in the pre-operative detection of accessory spleen is low. In this group, 28 patients underwent surgery and 12 accessory spleens were removed. Another finding that showed low sensitivity of SSS was detection of only 3 accessory spleens out of 12 (>1 cm) by scintigraphy. This may reflect low uptake by the accessory spleen in the presence of the spleen, the smaller size of accessory spleens than the resolution limits of gama cameras and not being able to assess the neighboring area due to intensive uptake of the spleen (superposition of planar images and a noise due to backprojection in SPECT images). Indeed, 9 accessory spleens (<1.5 cm) not detected by scintigraphy were located in the hilar region. This study showed that SSS failed to detect an accessory spleen in the hilar area when the accessory spleen was <1.5 cm in size in non-splenectomized patients. In a study investigating the contribution of a gamma probe in laparoscopic splenectomy, it was reported that of 17 patients, 2 had accessory spleens and pre-operative scintigraphic imaging was negative in both cases (15).

Selective spleen scintigraphy was positive and negative in 57% and 43% of patients in the SP group, respectively. The positivity rate of 57% might reflect that most of the patients had crITP, in whom the platelet counts decreased after surgery and were selected as clinically suspected cases. According to Normand et al., the incidence of accessory spleens/splenosis varies between 16% and 67% in patients who have undergone post-traumatic splenectomies; the rate falls far lower in patients with splenectomies for non-traumatic reasons (16). The rate of accessory spleens/splenosis after laparoscopy has been reported to be 50% (17). Being able to identify the smaller foci by SSS as compared with the NSP group suggests that high positivity is not only due to selected cases. Increased uptake of accessory spleens/splenosis tissue as a result of splenectomy may have increased the sensitivity. In our patient group, in addition to the scintigraphic findings noted on conventional imaging methods, a single focus could be detected. These findings showed that SSS is a sensitive technique for detection of accessory spleens/splenosis in splenectomized patients.

Of the patients in both groups with histologic verification, none had pathology reports apart from accessory spleens/splenosis. The results were consistent with the other imaging methods. These findings show that the specificity of SSS is higher in detecting accessory spleens/splenosis. No cases suggesting false positivity have been reported in the literature.

The differences observed between the SSS and other imaging methods on the basis of lesion number have been attributed to the very small size of adjacently located lesions. An increase in resolution and the new contrast agents raise the frequency of detection of accessory spleens by radiologic examinations (18). Computerized tomography and MRI are highly sensitive in detection of an accessory spleen in the usual locations; however, in the presence of multiple splenosis in different areas, detection of all lesions with a single injection of contrast agent and a single shot may be challenging. It is one of the advantages of SSS that many other areas could be scanned with a single injection (19). Indeed, our patient in whom the foci of splenosis in the pelvic region were detected by SSS is a good example.

SPECT imaging is a technique with a lower resolution compared with planar imaging. However SPECT increases the contrast in the depths of tissues and provides a three-dimensional view by eliminating the problem of superposition of tissues that occurs in planar imaging. Our study showed no difference between two techniques. This may be related to the fact that the advantage of increase in contrast in the depth of tissues provided by SPECT does not have a significant contribution, as SSS has a high target-to-background ratio. Another reason may be the fact that the hilar region that could not be adequately assessed by planar images because of the superposition and where the accessory spleen is often located might not be sufficiently evaluated by SPECT imaging because backprojection-related noise artifact is more intense in concave area of the spleen. For other areas, acquisition of planar images at many different angles have eliminated the problem of superposition. In our study LPO, LLAT and LAO images led to estimates of similar localization to SPECT images.

The foci of splenosis were better shown by SPECT/CT than planar imaging in a case report published by Alvarez et al. (20). SPECT/CT was shown to be more sensitive than planar imaging in a study of 7 cases published by Horger et al. (21); however, planar images were only acquired in the anterior-posterior positions. In a study by Phom et al. using Tc99m-anti-D IgG opsonized erythrocytes, there was no difference between SPECT and planar imaging with respect to detecting accessory spleens/splenosis (22).

No contribution of SPECT to planar imaging suggests a need for additional planar imaging like LPO, LLAT, LAO, which lasts about 30 min; however, instead of planar imaging, only SPECT imaging may be preferable. SPECT/CT is considered to be the better choice because the majority of patients will undergo surgery and require pre-operative anatomic imaging. The contribution of CT to localization is evident. The effect of CT used for attenuation correction on the sensitivity of SSS is unknown. Schillaci et al. suggested the potential superiority of SPECT/CT in detecting accessory spleen/splenosis (23).

## CONCLUSION

SSS has a high specificity in the detection of accessory spleens/splenosis. The sensitivity of SSS for detection of accessory spleens/splenosis is higher after splenectomy, and lower before splenectomy. When compared with other studies, SSS allows scanning accessory spleens/splenosis in unusual localizations without the need for additional radiation dose and re-injection. No additional contribution of SPECT to planar imaging was determined in acquiring images in multiple positions like lateral and oblique.

## Figures and Tables

**Table 1:**
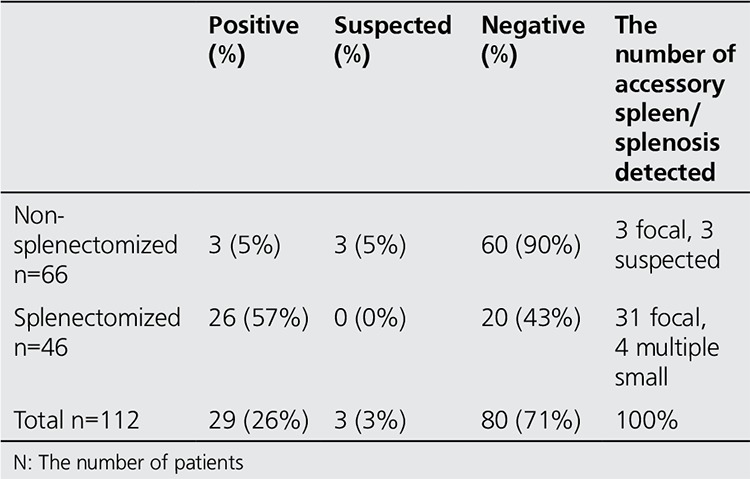
Distribution of the results of selective spleen scintigraphy in patients with and without splenectomy

**Table 2:**
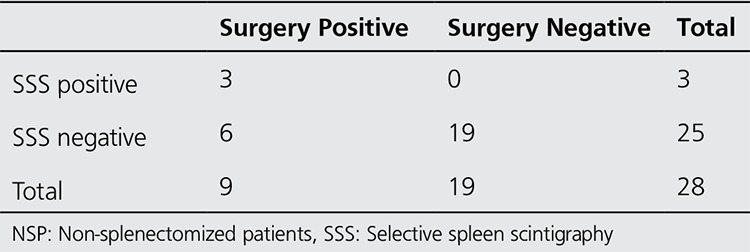
Comparison of the results of selective spleen scintigraphy with surgical results in the non-splenectomized patients group

**Table 3:**
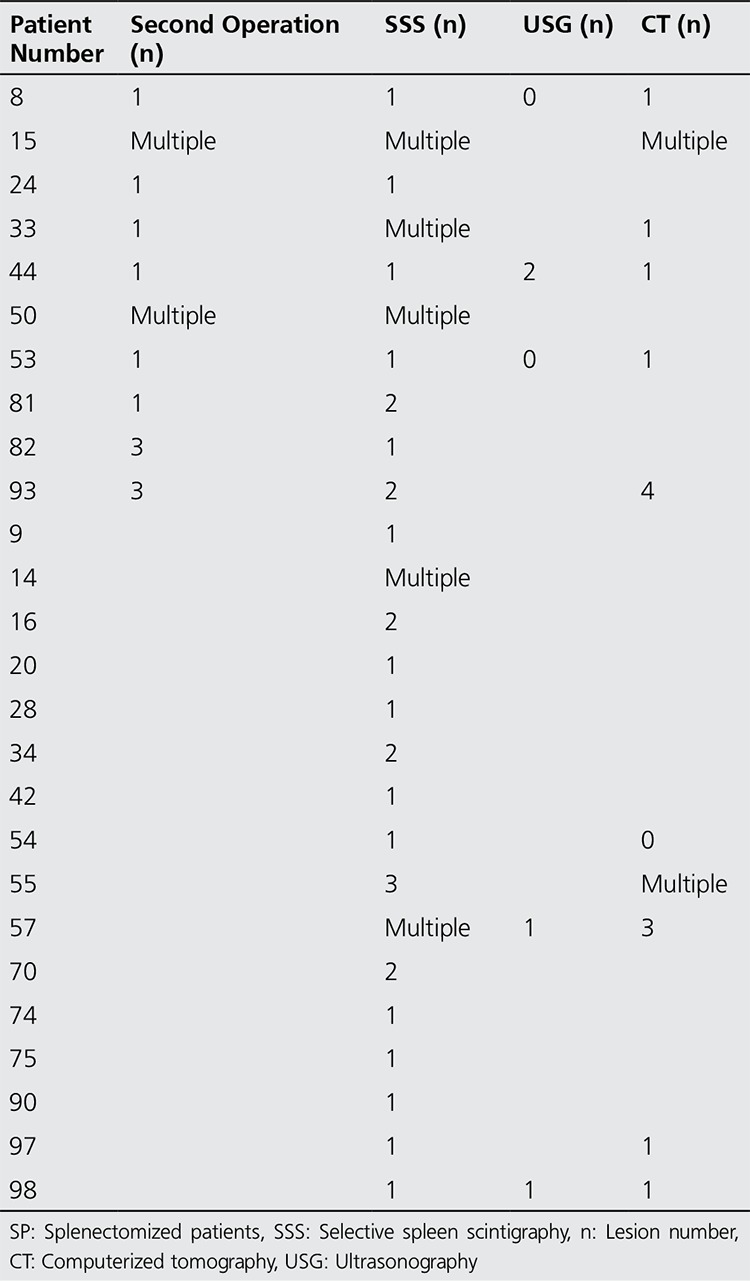
Comparison of selective spleen scintigraphy, ultrasonography, computerized tomography, and second operation results in splenectomized patients group

**Table 4:**
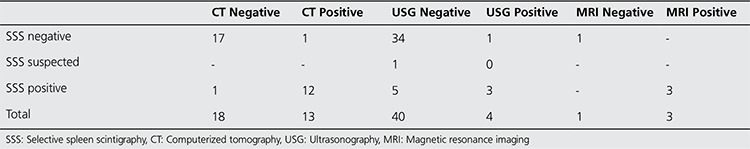
A comparison of the results of other imaging techniques to those of selective spleen scintigraphy

**Table 5:**
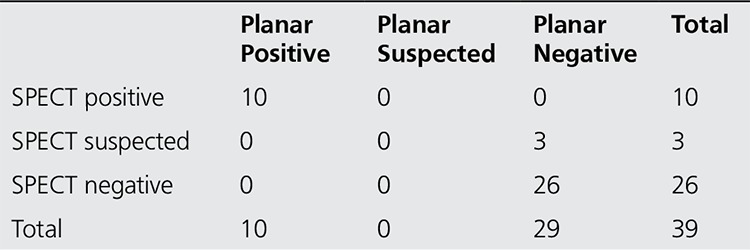
Comparison of planar with SPECT images
